# Impact of anti-TB drugs on modulations of T-cell-receptor-mediated signaling events in TB pleurisy patients

**DOI:** 10.3389/fimmu.2025.1645200

**Published:** 2025-12-01

**Authors:** Bhawna Sharma, Beenu Joshi, Santosh Kumar

**Affiliations:** 1 Department of Immunology, Indian Council of Medical Research (ICMR)-National JALMA Institute for Leprosy and Other Mycobacterial Diseases, Agra, India; 2 Department of TB and Other Chest Diseases and Respiratory Medicine, S.N. Medical College, Agra, India

**Keywords:** tuberculosis, cytokines, T cells, interferon gama (IFNγ), activation

## Abstract

Immunity in tuberculosis (TB) infection is complex as *Mycobacterium tuberculosis* (MTB) is a highly adaptive pathogen and may escape the immune defense through various ways. During MTB infection, immune modulation involves the activation and regulation of various immune cells and signaling pathways to mount an effective defense against the pathogen while minimizing immune pathology. Host pathogen interactions in TB are complex as MTB is a pathogen that is able to adapt and survive and may escape the immune defense through various ways. The limitations of BCG vaccine have energized researchers to identify alternative vaccines for TB. For the rational design of new efficacious and safe vaccines against TB, advanced knowledge of protective and pathological immune responses in TB is needed. It has been well established that the existing anti-TB treatment (ATT) induced an enhanced production of IL-2 and IFN-γ by T cells. This study explores modulations in the activation/phosphorylation of T-cell signaling molecules in the peripheral blood of TBP patients following 6 months of treatment. We reviewed existing evidence on TCR signaling alterations in TB and propose mechanisms by which treatment influences the activation of intracellular calcium mobilization and ZAP-70, PKC-theta, and MAPK activation, which is finally impacting T-cell function by regulating the production of cytokines and impacting the immune control of MTB. Our findings suggest that while treatment reduces bacterial burden, residual immune dysregulation in T-cell activation pathways may persist, influencing long-term T-cell responses. Further studies are needed to fully elucidate these changes and their implications for relapse prevention and therapeutic strategies.

## Introduction

Tuberculosis (TB) is a global emergency and remains a major bacterial cause of mortality. Although TB is predominantly a disease of lung parenchyma, i.e., PTB, it can involve a number of extrapulmonary sites. The resurgence of TB worldwide has intensified research efforts directed at examining the host defense and pathogenic mechanisms operative in TB infection. *Mycobacterium tuberculosis* (MTB), the etiological agent of TB, is a leading cause of death worldwide. An estimated 10.8 million people fell ill with TB in 2023, and 1.25 million people died (1). A hallmark of MTB infection is the ability of most healthy adults to control the infection through acquired immunity, in which antigen-specific T cells and macrophages arrest the growth of MTB bacilli and maintain control over persistent bacilli (2). The vaccine which is available against MTB is bacillus of Calmette and Guerin (BCG). BCG is a live vaccine prepared from attenuated strains of *M. bovis*. Although the BCG vaccine has been in use since 1921 and approximately 3 billion doses have been administered, its efficacy continues to be debated (3). Several trials have been performed to assess the efficacy of the vaccine, and the results vary. Thus, new vaccines and immunotherapeutic strategies are urgently required to improve TB control efforts.

BCG has antigen cross-reactivity with MTB and the NTMs, with either masking or blocking effects that might be the reason for the low efficacy of BCG (4). The use of MTB-specific antigens as vaccine candidates is expected to overcome the problems of blocking or masking effects (5). Ag85A and ESAT-6 are used to study TB immune responses because they are MTB-specific, immunodominant antigens recognized by T cells during infection and are associated with protective immunity in animal models (6). Ag85A and ESAT-6 are widely utilized antigens in TB research as they are highly immunogenic proteins from MTB that reliably elicit robust cellular and humoral immune responses in infected individuals, making them ideal for investigating host immunity, vaccine efficacy, and diagnostic markers. Their use allows researchers to discriminate between active TB and latent infection, identify specific T-cell populations, and evaluate the potential of new vaccines and diagnostic tools by measuring cellular responses like IFN-γ production.

Vaccine development depends on a comprehensive understanding of the host–pathogen interactions during MTB infection. The host immune responses play a central role in the establishment of long-term immune responses, which effectively helps in infection clearance. Conventional anti-TB therapy acts by interacting with the host immune system and employing it for the successful eradication of MTB (7). National TB treatment guidelines strongly recommend using a patient-centered case management approach—including directly observed therapy short-course (DOTS) for treating active TB patients, and it can affect the immune system in multiple ways, but the effects of the anti-TB drugs on the host immune system are not well elucidated to date. Few studies suggest that the TB drugs can lower the overall immune response, potentially impacting the body’s ability to fight off the infection or prevent recurrence. Toufis et al. observed that isoniazid (INH) hampers Th cells and makes the host more susceptible to reactivation (8). Host immune responses are known to target proteins that are secreted by MTB consequently; these proteins have been targeted for the development of vaccines and immunodiagnostics (9). During infection with MTB, innate mechanisms help to control bacillary spread, but T-lymphocyte recruitment to the lung is required to contain the infection in granulomas (10). It has been noted that protective immunity requires the generation of T-helper 1 (Th1) cytokine responses and IFN-γ, which activates macrophages to inhibit mycobacterial growth (11). Persons with mutations linked to IFN-γ signaling have increased susceptibility to mycobacterial infection and disseminated infection after BCG vaccination (12). Besides that, T cells from patients with TB produce less IFN-γ than those from persons with latent MTB infection (13), and IFN-γ production is lowest in patients with the most severe manifestation of TB (14). This shows that a better understanding of mechanisms for the regulation of IFN-γ production in TB patients might be crucial to develop new strategies to combat TB.

T lymphocytes respond to antigenic stimulation that drives their own proliferation, increasing the number of responsive T lymphocytes and amplifying the immune response. The key element in the initiation of T-cell activation is recognition by the T-cell receptor (TCR) of MHC–peptide complexes on antigen-presenting cells. The TCR consists of a mostly extracellular ligand binding unit, a predominantly intracellular signaling unit, the CD3 complex, and the homodimer of zeta chain. There are several signal transduction pathways associated with T-cell activation. Mitogen-activated protein kinases (MAPKs) are involved in many aspects of immune responses, including the initiation of innate immunity, activation of adaptive immunity, and termination of immune responses through cell death and regulatory T cells (15). MAPKs are essential for macrophage activation and regulation of IFN-γ production by ERK and p38 MAPK signaling pathway and through SLAM costimulation, which has been reported in TB (16). Moreover, MAPKs phosphorylate and activate downstream molecules, resulting in T-cell activation, proliferation, and differentiation into T-helper phenotypes (17). The signals triggered by TCR and CD28 co-stimulatory molecules induce membrane translocation and kinase activation of PKC-θ, leading to the subsequent activation of NF-κB and AP-1 (18).

The studies involving TCR-mediated mechanism of T-cell activation in TB using patient samples are still inconclusive. It has been previously observed that T cells from human TB patients had decreased the expression of CD3-ζ, a key signaling domain of the TCR/CD3 complex (19). Wang et al. have shown that the potent T-cell antigen ESAT-6 can directly suppress IFN-γ production in CD4+ T cells (20). Mahon et al. (21) reported that MTB cell wall glycolipids directly inhibit polyclonal murine CD4+ T-cell activation by blocking ZAP-70 phosphorylation, and later they extended their study by reporting ManLAM-induced inhibition of TCR signaling by interference with ZAP-70 (Zeta-chain-associated protein kinase 70) and Lck and LAT phosphorylation in antigen-specific murine CD4+ T cells and primary human T cells (22). Palma-Nicholás et al. (23) reported T-cell down-modulation of the MAPK–ERK1/2 pathway in total spleen cells from naive BALB/c mice by the cell-surface lipid di-O-acyl-trehalose (DAT). Inhibition of IFN-γ production through p38 MAPK pathway by ESAT-6 has been reported in T cells from healthy individuals (24). There are only a handful studies in India on the mechanism of T-cell activation in TB patients; most studies focus on pulmonary TB, with less known about T-cell dynamics in extrapulmonary TB. In our previous work, we have shown the effect of MTB antigen stimulation on the activation of MAPKs-ERK1/2 and P-38 in TB patients and healthy controls, and we found decreased phosphorylation of ERK1/2 and p38 after MTB antigen treatment in TB patients, while only ERK1/2 phosphorylation was inhibited in healthy individuals. In addition to this, we also observed that binding of transcription factors NFAT and NFκB was also altered by MTB antigens (25). We also studied modulations in T-cell signaling events in Jurkat T cells in our previous study (9).

TB pleurisy (TBP) is a naturally occurring type of MTB infection and thought to be a good model system to study cell-mediated responses at the infection site compared with peripheral blood. Tuberculous pleural effusion is enriched with CD4 lymphocytes (26). TBP is characterized by robust T-cell responses at the site of infection, with TCR-mediated signaling playing a critical role in immune activation. There is a previous study which showed that among pleural fluid lymphocytes, natural killer (NK) cells are a major source of IFN-γ production in a mechanism enhanced by IL-12, dependent on calcineurin, p38, and ERK pathways, and these cells are able to directly recognize MTB antigens (27). Our previous published data compared the activation of T-cell signaling events in the blood and pleural fluid of the same TB pleurisy patients, and we observed increased calcium influx as well as increased activation of ZAP-70, PKC-theta, and MAPKs in pleural fluid compared to blood. Our findings gave strong evidence that TCR-mediated T-cell activation could be involved in T-cell dysfunctioning during the progression of the disease and also could be responsible for Th 1 dominance at the local disease site in patients with TBP (28). However, the impact of 6 months of standard treatment on these pathways in circulating T cells remains underexplored. The present study examines modulations in TCR-mediated intracellular calcium levels, activation of ZAP-70, PKC-theta, and MAPK signaling focusing on potential changes in ERK1/2 and p38 activation in the blood of TB pleurisy patients in a longitudinal study where patients have been followed up after 6 months of treatment. By addressing the gaps in our understanding of T-cell dynamics, we can advance toward more effective TB management and prevention strategies.

## Methodology

### Study population

A hypothetical cohort of 15 TB pleurisy patients (*n* = 15) with ages between 18 and 60 years was the study population in this study. All patients included in the study were enrolled from the OPD of the Department of Tuberculosis and Chest Diseases, S.N. Medical College Agra. These 15 patients were followed up for 6 months after receiving treatment. The patients received the standard 6-month DOTS (out of 15 patients, only 10 patients could be followed up). Blood samples were collected at baseline before starting the treatment) and after receiving 6 months of treatment. The demographic details, PPD stattus and BCG vaccination status of all study participants have been mentioned in **Table 1**. We collected the detailed medical history of each patient, and all patients underwent detailed physical examination. The diagnosis of TB pleurisy was done by pleural fluid analysis—a positive result for *M. tuberculosis* by culture or nucleic acid amplification in pleural fluid sample. Patients with a positive test for human immunodeficiency virus, pregnant women, and those with the presence of concurrent infectious diseases were excluded.

**Table 1:** Demographic data of the study participants.

Characteristics	TBP	TBP-PT
Patients	15	10
Age Median Range (lower–upper)	43 (32.84–47.16)	40 (28.54–52.89)
Sex Male Female	11 (73.33%) 4 (26.66%)	7 (70%) 3 (30%)
BCG Vaccinated Non-vaccinated	11 (73.33%) 4 (26.66%)	7 (70%) 3 (30%)
PPD status Positive Negative	7 (46.67%) 8 (53.33%)	4 (40%) 6 (60%)

TBP, TB pleurisy patients; TBP-PT, TB pleurisy patients after 6 months of treatment.

This study has taken approval from the Human Ethics committee of ICMR-National JALMA Institute for Leprosy and Other Mycobacterial Diseases, Agra (IHEC). Informed written consent was also obtained from all study subjects.

### Sample collection and peripheral blood mononuclear cell preparation

Peripheral blood was collected from the patients at baseline/at the time of enrollment in the study and after 6 months of treatment. Blood samples were collected in heparinized vials, and PBMCs were separated using Ficoll-Hypaque density gradient centrifugation method. After washing of the buffy coat of PBMC, the cells were suspended in RPMI 1640 tissue culture medium (SIGMA, USA supplemented) with 2 mM L-glutamine, antibiotic–antimycotic solution (Sigma, USA), and 10% heat-inactivated human AB serum (MP. Biomedicals, India). Cell viability ≥95% was determined by Trypan blue exclusion test. The cells were plated in 24-well culture plates, and the cultures were maintained in a humidified 5% CO_2_ incubator at 37°C.

### Chemicals and antigens

The mouse IgG anti-human pure CD3 antibody (clone UCHT1), ionomycin, goat anti-mouse-IgG antibody, sodium fluoride (NaF), sodium orthovanadate, anti-protease cocktail, and Bradford reagent were procured from Sigma, USA. Fura-2/AM, used for intracellular calcium studies, was procured from Calbiochem, USA. Antibodies-anti-human CD3 (clone OKT-3) and anti-human CD28 (clone CD28.2) for T-cell activation were procured from eBiosciences, USA. Cell The extraction buffer was a ready-to-use lysis buffer from Invitrogen, Thermo Fisher Scientific Inc., USA. The antibodies for studying the activation of various molecules—PhosphoZAP-70, PhosphoERK1/2, phospho p38, phospho PKC-θ, β-actin, and goat polyclonal IgG anti-mouse horse radish peroxidase (HRP) conjugated antibody were procured from Cell Signalling Technology (CST), USA. The reagents for performing enhanced chemiluminescence assay (ECL) were procured from Millipore, USA. Lyophilized MTB antigens (ESAT-6 and Ag85A) were procured from BEI Research Resources Repository funded by the National Institute of Allergy and Infectious Diseases and managed by ATCC, USA. All antigens were dissolved in filtered phosphate-buffered saline (PBS), pH 7.4, to make a 1-mg/mL concentration.

### Intracellular Ca^2+^ mobilization estimation by spectrofluorimetery

For studying the intracellular calcium levels in the cells of patients before and after treatment, PBMCs at the concentration of 5 × 10^6^/mL per reaction were rested for at least 2 h in a 37°C CO_2_ incubator before stimulation. After resting, the cells were stimulated with appropriate doses of MTB antigens in a 37°C CO_2_ incubator; after incubation, the cells were washed with PBS. The cells were incubated with Fura-2/AM at 1 μM for 30 min at 37°C in a loading buffer with pH 7.4, containing [NaCl, 110 mM; KCl, 5.4 mM; NaHCO_3_, 25 mM; MgCl_2_, 0.8 mM; KH_2_PO_4_, 0.4 mM; HEPES, 20 mM; Na_2_HPO_4_, 0.33 mM; and CaCl_2_ 1.2 mM. After incubation with Fura, the cells were washed three times and finally suspended in loading buffer. Intracellular calcium levels were measured as per the previously published protocol (29, 30). Fluorescence intensities were measured in ratio mode using Varian ECLIPSE spectrofluorometer equipped with a fast filter accessory [Varian at 340 nm and 380 nm (excitation filters) and 510 nm (emission filter)]. The cells were stirred continuously throughout the experiment. For anti-CD3-stimulated calcium studies, 10 µg/mL of pure anti-CD3 (Clone UCHT1) was added to cuvette after stabilization of basal levels of cytosolic calcium. For the measurement of *F*_max_, ionomycin at a concentration of 5 µM was added to the cuvette, and for *F*_min_ 2 mM MnCl_2_ was added.

The intracellular concentrations of free Ca^2+^ [Ca^2+]^i] were calculated by using the following equation: [Ca^2+^]i = Kd × (*R* − *R*_min_)/(*R*_max_ − R) × (Sf2/Sb2). A value of 224 nM for Kd was added into the calculations. *R*_max_ and *R*_min_ values were obtained by the addition of ionomycin (5 μM) and MnCl_2_ (2 mM), respectively. All experiments were performed at 37°C.

### Stimulation of cells with MTB antigens and activation of T cells

PBMCs at a concentration of 5 × 10^6^/mL per reaction were used for lysate preparation. The cells were rested for at least 2 h in a 37°C CO_2_ incubator. After resting, the cells were stimulated overnight with appropriate doses of MTB antigens in 37°C CO_2_ incubator. After overnight stimulation with MTB antigens, the cells were activated with plate-bound anti-CD3 and anti-CD28 antibodies at 2 μg/mL each. The antibody-coated plates were prepared by coating the wells with goat anti-mouse IgG for 1 h at 37 °C; after washing twice with PBS, the plates were coated with both anti-CD3 and anti-CD28 at a concentration of 2 μg/mL for 1 h at 37°C in a humidified atmosphere of 5% CO_2_. The antigen-stimulated cells were then added to the antibody-coated wells and incubated overnight at 37°C in a humidified atmosphere of 5% CO_2_. Few cells were left untreated with antigens, and few that were left untreated with CD3 and CD28 antibodies also were used as control.

### Estimation of activation of ZAP-70, PKC-theta, and MAPK molecules by western blotting and enhanced chemiluminescence assay

PBMCs were stimulated with MTB antigens and activated with CD3/CD28 antibodies. After activation with CD3/CD28 antibodies, the cells were removed from the plate and washed with chilled PBS, and then 50 μL of cell extraction buffer (supplemented with 1 mM PMSF and protease inhibitor cocktail) was added. The cell pellets were lysed in cell extraction buffer for 30 min; the cells were kept on ice, with intermittent vortexing at every 10-min interval. After incubation in lysis buffer, centrifugation was done at 13,000 × *g* for 10 min at 4 °C. The cell lysates were transferred in fresh tubes and used immediately or stored at −80°C. The protein concentration was estimated by Bradford protein estimation method. Denatured proteins (35 μg) were separated by SDS-PAGE (10%) and transferred to polyvinylidine difluoride (PVDF) membrane. Immunodetection of phosphorylated forms of ZAP-70, PKC-θ, Erk1/2, and p38MAPK was done using 1:1,000 dilution of phospho-specific antibodies for ZAP-70, PKC-θ, Erk1/2, and p38MAPK in 5% BSA TBS. The membranes with phosphorylated primary antibodies were incubated at 4°C overnight. After overnight incubation, the membrane was washed thrice with TBST (TBS with 0.05% Tween-20). Then, the PVDF membranes were treated with HRP-conjugated secondary antibody, and peroxidase activity was detected with ECL reagents. Equal loading of the proteins was confirmed after stripping the membrane and reprobing it for total forms of β-actin. Densitometric analysis of bands was performed using Quantity One™ software (Bio-Rad, Hercules, USA).

### Analysis of IFN-γ- and IL-2-producing T-cell population by flow cytometry

To estimate the frequency of IFN-γ- and IL-2-producing T cells, we performed intracellular cytokine staining. PBMCs were stimulated overnight with appropriate doses of MTB antigens in a 5% CO_2_ incubator at 37 °C. Brefeldin A (1 μg/mL) (BD Biosciences, CA, USA) was added in the culture 16 h before the completion of incubation. The surface markers conjugated with fluorochrome for T helper cell staining were CD3 APC (cat. no. 555335 UCHT1 clone) and CD4 PerCPCy5.5 (cat. no. 341654 SK3 clone) (BD Biosciences, CA, USA). The cells were incubated at 4°C for 30 min for surface marker staining. Then, the cells stained with surface markers were fixed and permeabilized by treating the cells using Cytofix/Cyto Perm Buffer (BD Biosciences, CA, USA). After permeabilization, the cells were distributed in two separate tubes and were stained with antibodies for intracellular cytokines—IFN-γ FITC (cat. no. 554700 B27 Clone) and IL-2 FITC (cat no. 554565 MQ1-17H12 clone) (BD Biosciences, CA, USA). Isotype controls were also used for proper gating. The cells were suspended in a staining buffer containing 2% paraformaldehyde and acquired by using a flow cytometer (FACSAria, BD Biosciences, USA). Analysis of the acquired data was done using FlowJo v7.6 software (FlowJo LLC, OR, USA).

### Gating strategy

The gating strategy for the CD3+CD4+ cells producing IFN-γ and IL-2 was as follows: The lymphocytes were gated on a forward scatter (FSC)/side scatter (SSC) dot plot. Furthermore, lymphocytes were gated to determine CD3+ and CD4+ populations of T helper cells. Isotype controls were also used for proper gating. Double-positive CD3+CD+4 T helper cells were also gated to estimate the frequency of IL-2- and IFN-γ-producing cells.

### Statistical analysis

Data were presented as mean ± SEM, and comparisons of pre- and post-treatment and the effect of various antigen stimulations on the expression of various molecules on a patient’s blood were performed using the nonparametric Mann–Whitney *U*-test *t*-test. Analysis was done with Prism 5.0 software (GraphPad, La Jolla, CA, USA). Moreover, *p*-values less than 0.05 were considered as statistically significant.

## Results

### Differential changes in intracellular Ca^2+^ mobilization in the blood of TB pleurisy patients after 6 months of follow-up

**Figure 1:**
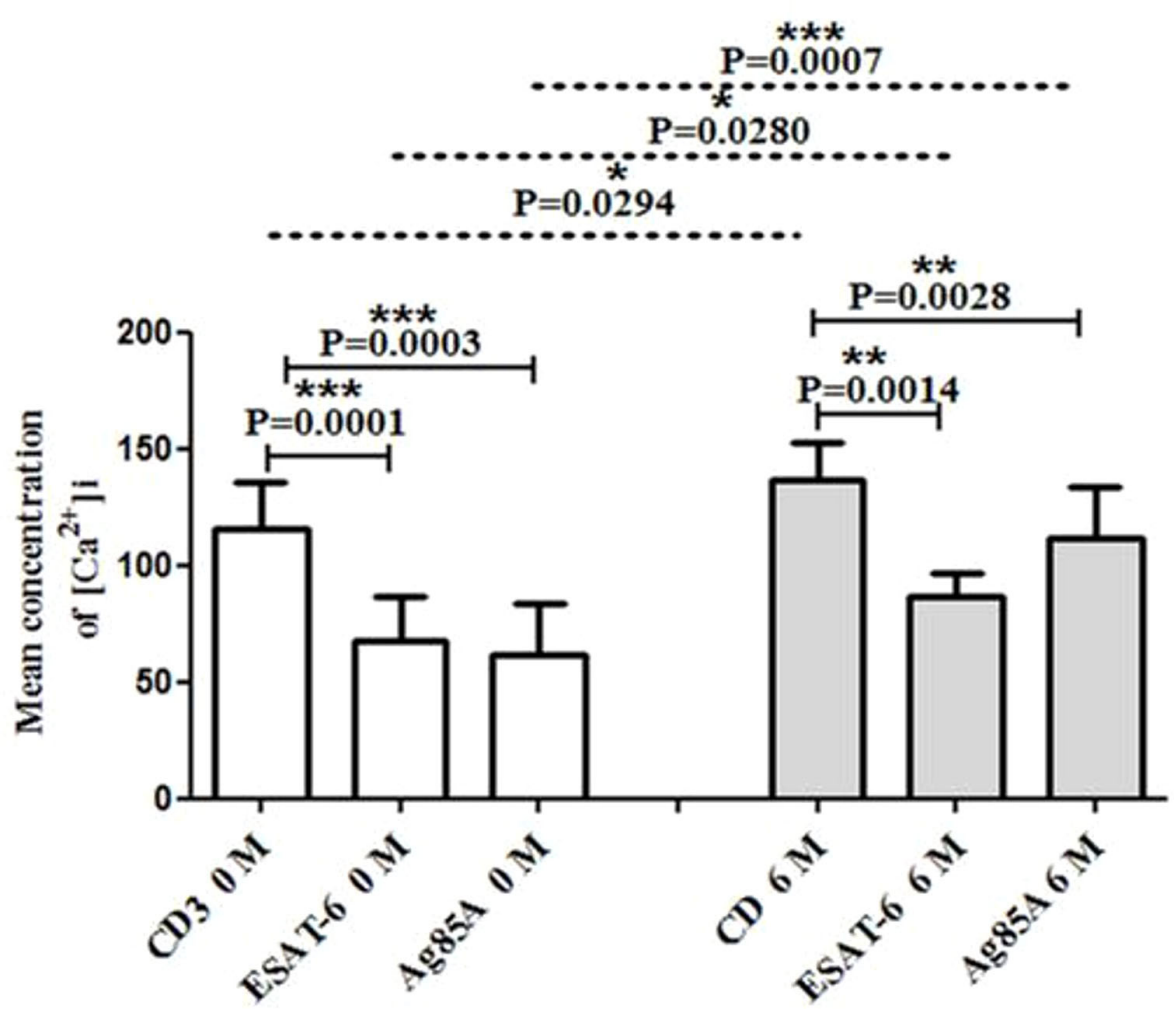
Modification in CD3 induced free intracellular calcium concentration in blood after MTB antigen (ESAT-6 and Ag85A) stimulation. The Fura-2AM-loaded cells (PBMCs) were used to study intracellular calcium levels, and fluorescence intensities were measured in ratio mode using Varian ECLIPSE spectrofluorometer as described in the materials and methods section. The bar diagrams show changes in intracellular calcium levels in CD3-treated cells. The effect of MTB antigens on CD3-stimulated calcium influx is shown in graphs of the blood of TBP patients at baseline (0M) and after 6 months of treatment (6M). The bar is showing mean ± SEM. *P < 0.05; **P < 0.005; *** p≤0.001.

In the present study, we estimated the effect of 6 months of anti-TB treatment on intracellular calcium levels in the blood of TBP patients, and we also investigated the effect of MTB antigens on intracellular calcium mobilization by spectrofluorimetry. We evaluated the effect of anti-CD3 antibody on the cells of patients’ blood pre-treated with optimum doses of MTB antigens (Ag85A and ESAT-6). We observed significantly higher intracellular calcium levels in CD3-triggered cells of patients after 6 months of treatment compared to the intracellular calcium levels in their blood at baseline/start of the treatment. We also observed significantly reduced CD3-triggered intracellular calcium in ESAT-6- and Ag85A-stimulated cells at baseline as well as after 6 months of treatment. Interestingly, the reduction was more in Ag85A-stimulated cells at baseline compared to after 6 months of treatment (**Figure 1**).

### Impact of 6 months of treatment and MTB antigen stimulation on ZAP-70 activation in the blood of TBP patients

**Figure 2:**
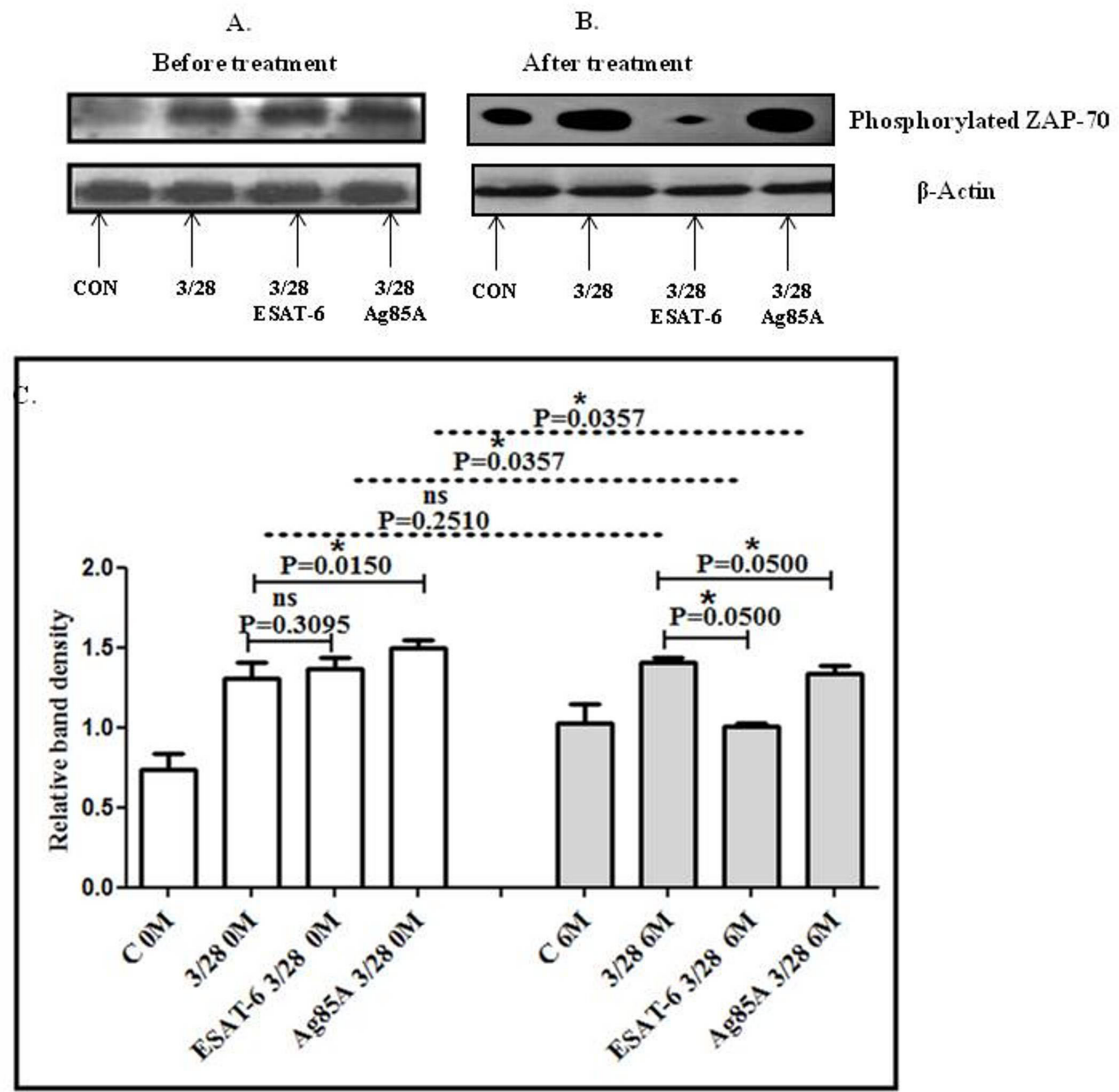
TCR/CD28-induced phosphorylation of ZAP-70 before and after MTB antigen stimulation in the blood of TBP patients at baseline (0M) and after 6 months of treatment (6M). PBMCs were activated with CD3 and CD28 antibodies after pretreatment with antigens. Few cells were left unstimulated and untreated with antibodies as negative control, while few cells were only activated with CD3 and CD28 without any antigen stimulation. Western blotting was done as mentioned in the materials and methods section. β-Actin antibody was used to conform equal loading. Densitometric analysis of phosphorylated ZAP-70 in the blood of a TBP patient at baseline before treatment **(A)** and after 6 months of treatment **(B)**. Relative band intensity values are expressed as mean ± SEM in bar diagrams **(C)**. A representative blot of one experiment with phosphorylated Zap-70 and β-Actin is shown. In Fig **(A)**, lane 1 shows before treatment (control) in blood, lane 2 is before treatment with anti-CD3 + anti-CD28-activated cells in blood, lane 3 shows before treatment with anti-CD3 + anti CD28-activated cells in blood with pre-treatment with ESAT-6, and lane 4 shows before treatment with anti-CD3 + anti-CD28-activated cells in blood pretreated with Ag85A. **(B)** Lane 1 shows control in blood after 6M of treatment, lane 2 shows anti-CD3 + anti-CD28-activated cells in blood after 6M of treatment, lane 3 shows anti CD3 + anti CD28-activated cells in blood after 6M of treatment with pre-treatment with ESAT-6, and lane 4 shows anti-CD3 + anti-CD28-activated cells in blood after 6M of treatment pretreated with Ag85A. **(C)** Densitometric analysis of phosphorylated ZAP-70 in blood at baseline and after 6M of treatment. Densitometric analysis was done, and the ratios of phosphorylated ZAP-70 to β-actin protein expression were expressed as arbitrary units. Statistical significance was determined using Mann–Whitney test. *P <.05; **P < 0.005; ***P < 0.005.

The effect of treatment and MTB antigen stimulation on ZAP-70 activation in CD3/CD28-stimulated cells of TBP patients was studied by performing Western blot. No significant difference in phosphorylated ZAP-70 levels was observed in CD3CD28-activated cells after 6 months of treatment compared to the baseline levels. However, phosphorylated ZAP-70 was observed to be significantly decreased in ESAT-6- and Ag85A-stimulated cells after 6 months of treatment. Altered activation of ZAP-70 was observed after MTB antigen stimulation in patients. It was significant with ESAT-6 but not significant with Ag65A at baseline. On the other hand, ESAT-6 and Ag85A both showed significantly increased phosphorylated ZAP-70 after 6 months of treatment (**Figure 2**).

### Impact of 6 months of ATT treatment and MTB antigen stimulation on the activation of protein kinase C-theta (PKC-θ) levels in TBP patients

**Figure 3:**
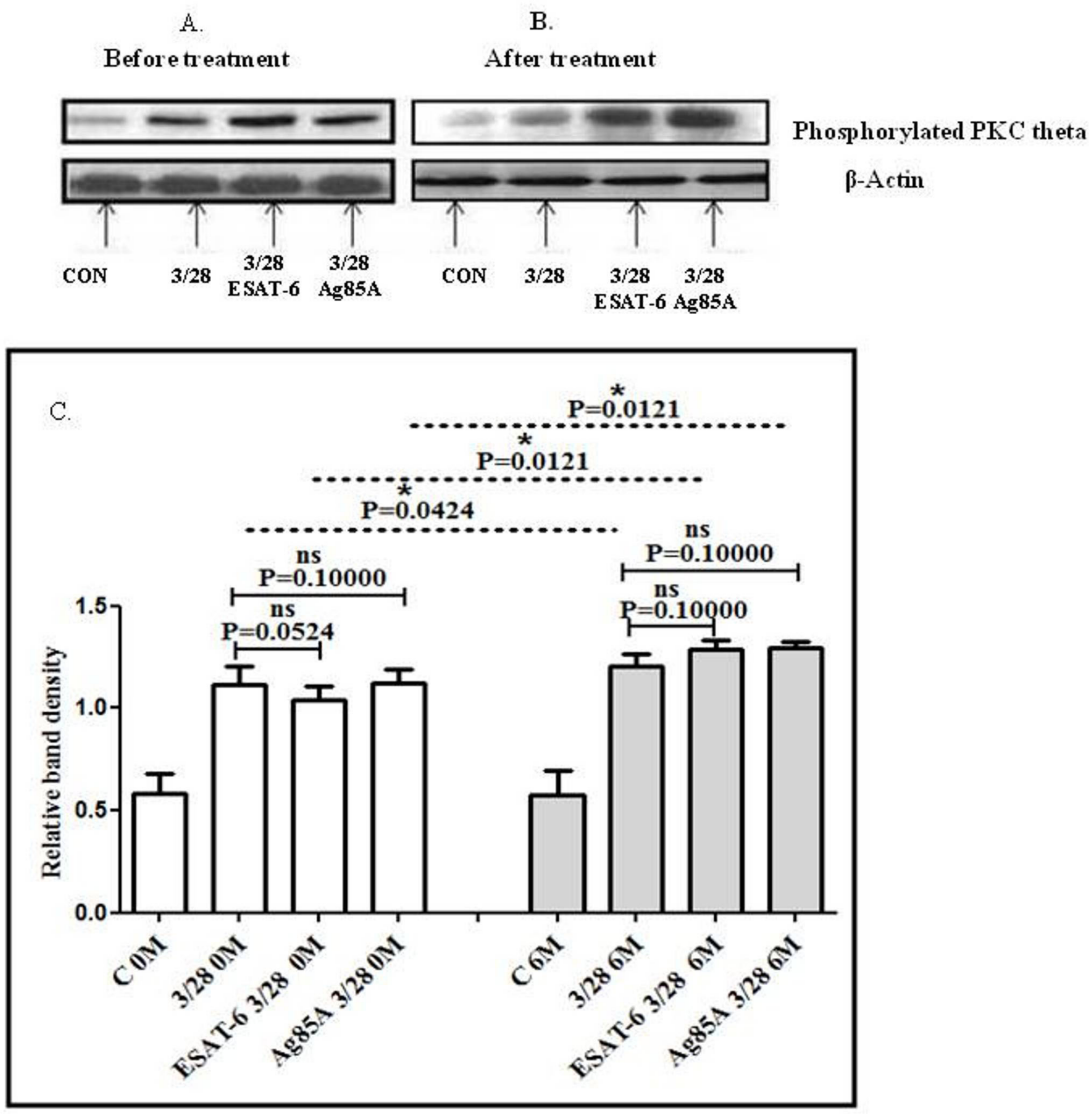
TCR/CD28-induced phosphorylation of PKC-θ before and after MTB antigen stimulation in the blood of TBP patients at baseline (0M) and after 6 months of treatment (6M). PBMCs were activated with CD3 and CD28 antibodies after pretreatment with MTB antigens. Few cells were left unstimulated and untreated with antibodies as negative control, and few cells were only activated without any antigen stimulation. Western blotting was done as mentioned in the materials and methods section. β-Actin antibody was used to conform equal loading. Densitometric analysis of phosphorylated PKC-θ in blood in a TBP patient at baseline before treatment **(A)** and after 6 months of treatment **(B)**. Relative band intensity values are expressed as mean ± SEM in bar diagrams **(C)**. A representative blot of one experiment with phosphorylated PKC-θ and β-actin is shown. **(A)** Lane 1 shows before treatment (control) in blood, Lane 2 shows before treatment with anti-CD3 + anti CD28-activated cells in blood, lane 3 shows before treatment with anti-CD3 + anti-CD28-activated cells in blood with pre-treatment with ESAT-6, and lane 4 shows before treatment with anti-CD3 + anti-CD28-activated cells in blood pretreated with Ag85A. **(B)** Lane 1 shows control in blood after 6M of treatment, lane 2 shows anti-CD3 + anti-CD28-activated cells in blood after 6M of treatment, lane 3 shows anti-CD3 + anti-CD28-activated cells blood after 6M of treatment with pre-treatment with ESAT-6, and lane 4 shows anti-CD3 + anti-CD28-activated cells in blood after 6M of treatment pretreated with Ag85A. **(C)** Densitometric analysis of phosphorylated PKC-θ in blood at baseline and after 6M of treatment. Densitometric analysis was done, and the ratios of phosphorylated PKC-θ to β-Actin protein expression were expressed as arbitrary units. Statistical significance was determined using Mann–Whitney test. *P <.05; **P < 0.005; ***P < 0.005.

Regarding the activation levels of PKC-θ in the blood of TBP patients before and after 6 months of ATT treatment and also to find out the effect of MTB antigen stimulation on CD3/CD28-induced PKC-θ activation, we performed Western blot. Significantly higher levels of phosphorylated PKC-θ were observed after 6 months of treatment. We observed a significantly altered activation of PKC-theta after MTB antigen stimulation in the blood of patients before as well as after 6 months of treatment. Significantly upregulated phosphorylated PKC theta levels were observed in patients after 6 months of treatment compared to the baseline (**Figure 3**).

### Effect of 6 months of treatment and MTB antigen stimulation on the activation of mitogen-activated protein kinase blood of TBP patients at baseline and after 6 months of ATT treatment

**Figure 4:**
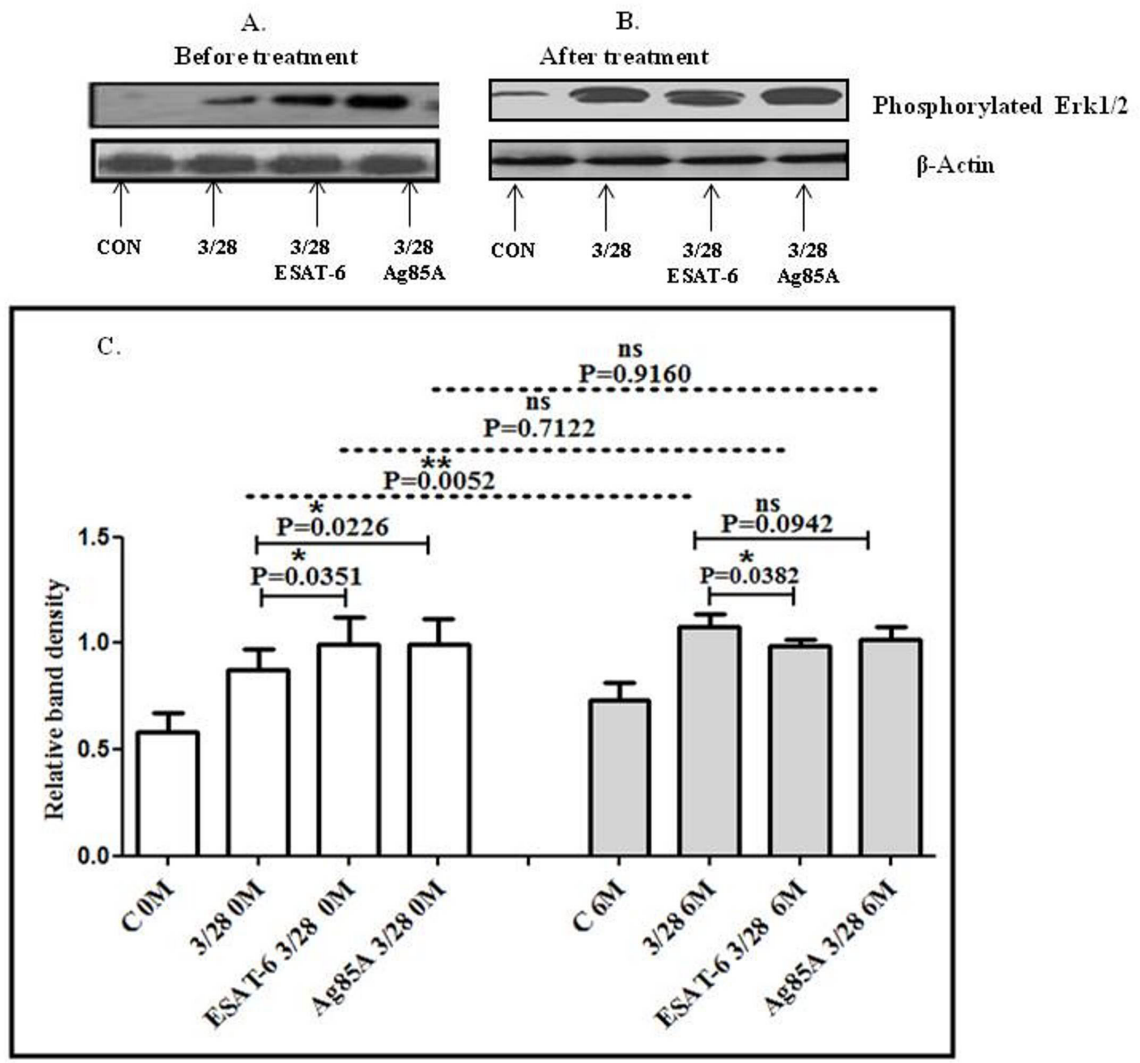
TCR/CD28-induced phosphorylation of Erk1/2 before and after MTB antigen stimulation in the blood of TBP patients at baseline (0M) and after 6 months of treatment (6M). PBMCs were activated with CD3 and CD28 antibodies after pretreatment with antigens. Few cells were left unstimulated and untreated with antibodies as negative control, and few cells were only activated with CD3 and CD28 without any antigen stimulation. Western blotting was done as mentioned in the materials and methods section. β-Actin antibody was used to conform equal loading. Densitometric analysis of phosphorylated Erk1/2 in blood in a TBP patient at baseline before treatment **(A)** and after 6 months of treatment **(B)**. Relative band intensity values are expressed as mean ± SEM in bar diagrams **(C)**. A representative blot of one experiment with phosphorylated Erk1/2 and β-actin is shown. **(A)** Lane 1 shows before treatment (control) in blood, lane 2 shows before treatment with anti-CD3 + anti CD28-activated cells in blood, lane 3 shows before treatment with anti-CD3 + anti-CD28-activated cells in blood with pre-treatment with ESAT-6, lane 4 shows before treatment with anti-CD3 + anti-CD28-activated cells in blood pretreated with Ag85A. **(B)** Lane 1 shows control in blood after 6M of treatment, lane 2 anti-CD3 + anti-CD28-activated cells in blood after 6M of treatment, lane 3 shows anti-CD3 + anti-CD28-activated cells in blood after 6M of treatment with pre-treatment with ESAT-6, lane 4 shows anti-CD3 + anti-CD28-activated cells in blood after 6M of treatment pretreated with Ag85A. **(C)** Densitometric analysis of phosphorylated Erk1/2 in blood at baseline and after 6M of treatment. Densitometric analysis was done, and the ratios of phosphorylated Erk1/2 to β-Actin protein expression were expressed as arbitrary units. Statistical significance was determined using Mann–Whitney test. *P <.05; **P < 0.005.

Western blotting was done to evaluate the differences in CD3/CD28-induced MAPK activation in the blood of TBP patients after 6 months of treatment. We also studied the effect of MTB antigen stimulation MAPK activation. We observed significantly higher levels of phosphorylated Erk1/2 in the blood of TBP patients after 6 months of treatment compared to baseline. After stimulation of cells with MTB antigens, altered activation of Erk1/2 was observed in cells at baseline as well as after 6 months of treatment. Increased phosphorylation of Erk1/2 was observed in MTB-antigen-stimulated cells at baseline, while significantly decreased levels of phosphorylated Erk1/2 were observed after treatment in MTB-antigen-stimulated cells (**Figure 4**).

**Figure 5:**
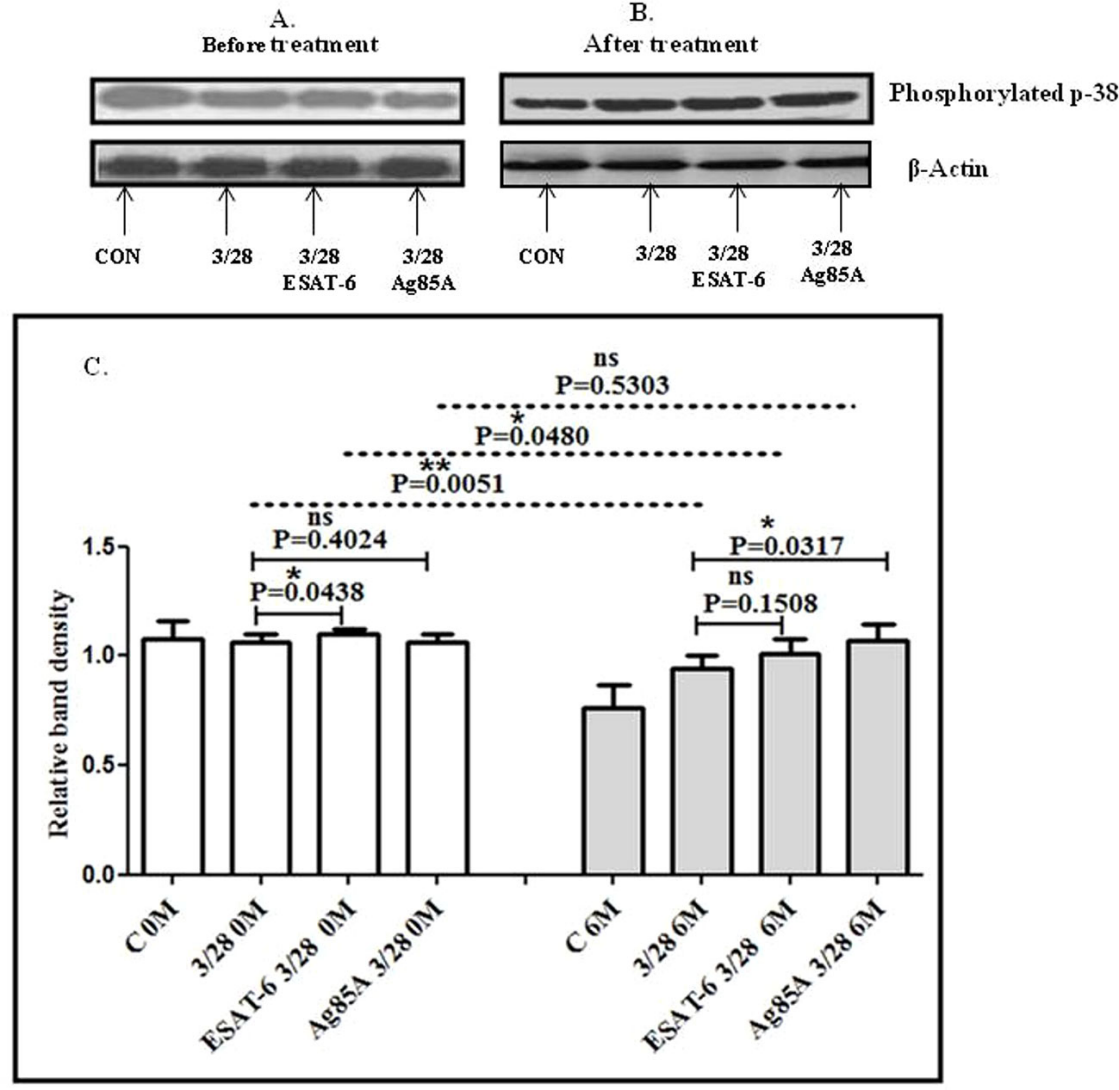
TCR/CD28-induced phosphorylation of p-38 before and after MTB antigen stimulation in the blood of TBP patients at baseline (0M) and after 6 months of treatment (6M). PBMCs were activated with CD3 and CD28 antibodies after pretreatment with *M. tuberculosis* antigens. Few cells were left unstimulated and untreated with antibodies as negative control, and few cells were only activated without any antigen stimulation. Western blotting was done as mentioned in the materials and methods section. β-Actin antibody was used to conform equal loading. Densitometric analysis of phosphorylated p38 in the blood of a TBP patient at baseline before treatment **(A)** and after 6 months of treatment **(B)**. Relative band intensity values are expressed as mean ± SEM in bar diagrams **(C)**. A representative blot of one experiment with phosphorylated p-38 and β-actin is shown. **(A)** Lane 1 shows before treatment (control) in blood, lane 2 shows before treatment with anti-CD3 + anti-CD28-activated cells in blood, lane 3 shows before treatment with anti-CD3 + anti-CD28-activated cells in blood with pre-treatment with ESAT-6, and lane 4 shows before treatment with anti-CD3 + anti-CD28-activated cells in blood pretreated with Ag85A. **(B)** Lane 1 shows control in blood after 6M of treatment, lane 2 shows anti-CD3 + anti-CD28-activated cells in blood after 6M of treatment, lane 3 shows anti-CD3 + anti-CD28-activated cells in blood after 6M of treatment with pre-treatment with ESAT-6, and lane 4 shows anti-CD3 + anti-CD28 activated cells in blood after 6M of treatment pretreated with Ag85A. **(C)** Densitometric analysis of phosphorylation of p-38 in blood at baseline and after 6M of treatment. Densitometric analysis was done, and the ratios of phosphorylated p-38 to β-actin protein expression were expressed as arbitrary units. Statistical significance was determined using Mann–Whitney test. *P <.05; **P < 0.005.

We observed a significantly decreased phosphorylation of p-38 in the blood of patients after treatment compared to baseline. Altered activation was observed after stimulation of cells with MTB antigens. ESAT-6 increased the phosphorylated p-38 levels at baseline and post-treatment; both were significant at baseline but not significant after treatment. Ag85A significantly increased p-38 phosphorylation in patients after treatment (**Figure 5**).

### Effect of treatment on the frequency of IFN-γ- and IL-2-producing T helper cells in the blood of TB pleurisy patients

**Figure 6:**
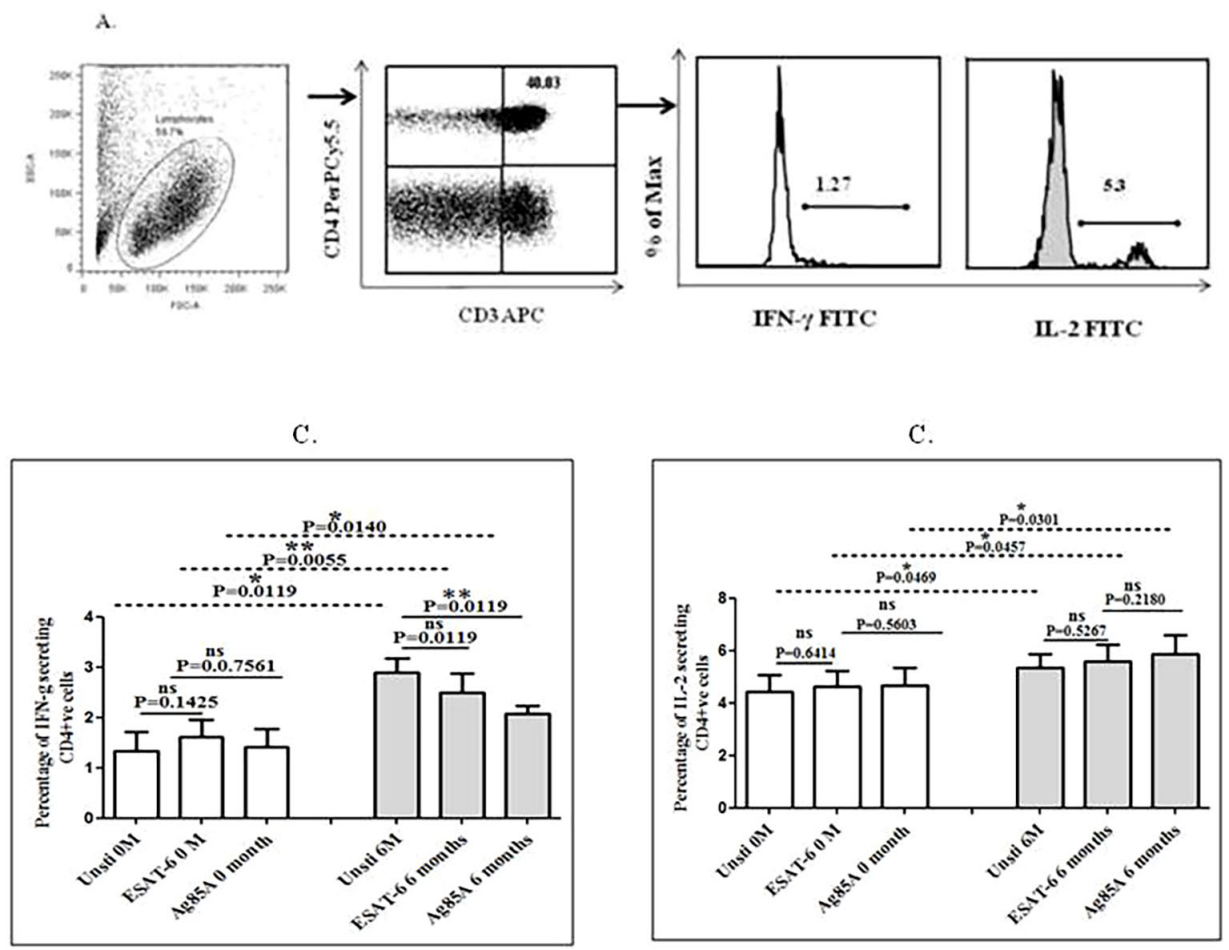
Intracellular cytokine staining was done to study the frequency of IFN-γ- and IL-2-producing cells in blood of TBP patients at baseline and after 6 months of follow-up. Cells were stimulated with MTB antigens overnight, and few cells were left unstimulated. Staining was done, and the expression of IFN-γ and IL-2 was observed on CD3+CD4+ T helper cells. Gating strategy for defining T helper cell type 1 (Th1) subset and representative flow cytometry plots of the study subject. **(A)** Gated lymphocytes on a forward scatter (FSC)/side scatter (SSC) were subsequently gated to determine CD3+CD4+ double-positive T helper cells. Further from CD3+CD4+gate, triple-positive population CD3+CD4+IFNγ+ and CD3+CD4+IL-2+ subsets were identified. Differential expression (in terms of percentage) of CD3+CD4+IFNγ+ and CD3+CD4+IL-2+ subsets was observed in the blood of TBP patients before and after treatment **(B)**. The comparison of percentage of CD3+CD4+IFNγ+ cell subset and **(C)** percentage of CD3+CD4+IL-2+ cell subset at baseline and after 6 months of treatment and also with MTB antigens stimulation was made using Mann–Whitney test (*p ≤ 0.05; **p ≤ 0.01).

Intracellular cytokine staining was done to study the frequency of IFN-γ- and IL-2-producing T helper cells in the blood of TBP patients at baseline and after 6 months of follow up. We observed a significantly higher percentage of IFN-γ and IL-2 production by CD4+ T cells after 6 months of treatment compared to baseline (**Figure 6**). A higher frequency of IL-2-producing CD3+CD4+ T cells was observed compared to the frequency of IFN-γ producing CD3+CD4+ T cells. We also observed an altered frequency of IFN-γ- and IL-2-producing CD3+CD4+ T cells after MTB antigen stimulations at baseline as well as after 6 months of treatment. A significantly increased percentage of IFN-γ- and IL-2-producing CD3+CD4+T cells was observed in ESAT-6- and Ag85A-stimulated cells after treatment compared to baseline (**Figures 6B, C**).

## Discussion

TB remains a major global health problem in India, with the highest number of TB cases that contributes significantly to the global TB mortality (1). The MTB-specific CD4+ Th1 cell response is crucial in the immunologic response to TB infection by recruiting and activating innate immune cells and producing cytokines such as IFN-γ (31). CD4+ T cells recognize mycobacterial antigens presented by antigen-presenting cells (APCs) via their TCR (32) and activate intracellular signaling pathways, including the MAPK, PI3K, and Ca^2+^ signaling pathways, which are crucial for T-cell activation (20, 25). These signaling events direct the activation of various transcription factors which are the key regulators of IFN-γ- and IL-2-production by Th1 cells. IFN-γ is a key cytokine to activate macrophages, which are essential to control bacterial infection. IL-2, on the other hand, is involved in T-cell proliferation, survival, and differentiation and also plays a role in regulating the immune response (33, 34). Herein our primary focus was to evaluate the impact of ATT on the activation of signaling pathway molecules. We studied intracellular calcium mobilization, CD3/CD28-triggered activation of ZAP-70, PKC-θ, and MAPKs in cells of TBP patients at baseline and after 6 months of treatment. We also evaluated the effect of ATT on T-cell function by studying the frequency of CD3+CD4+ producing IFN-γ and IL-2 cytokines after 6 months of treatment and at baseline. To date, there is no such study available that investigated the impact of ATT on the TCR-mediated T-cell signaling event. We also evaluated the effect of MTB antigens (ESAT-6 and Ag85A) on the activation levels of various T-cell signaling events.

It has been proved that elevated intracellular calcium levels are essential for T-cell activation and proliferation (35). We noticed significantly higher intracellular calcium levels in TBP patients after 6 months of treatment compared to baseline. In TB, ZAP-70 activation is essential for the T-cell-mediated immune response against TB (28, 36). Our results shows significant upregulation of ZAP-70 blood samples of TBP patients after 6 months of treatment compared to the blood of patients at the start of treatment.

PKC-θ is a key kinase that plays essential roles in controlling peripheral T-cell activation and preventing T-cell energy. The signals triggered by TCR and CD28 co-stimulatory molecules induce membrane translocation and kinase activation of PKC-θ, leading to subsequent activation of NF-κB and AP-1. In TB, the efficiency of T cells to respond against infection depends on the activation of transcription factors which eventually regulate cytokine production. PKC-θ-deficient T cells exhibit defects in T-cell activation, including reduced IL-2 production and impaired differentiation into inflammatory T cells (37).We observed a significant upregulation of PKC-θ activation in patients after 6 months of treatment compared to baseline. MAPKs are regulated by phosphorylation cascades and play an important role in T-cell activation. We observed that altered TCR induced MAPK activation in TBP patients at baseline and after 6 months of ATT. We observed upregulated ERK1/2 activation after treatment, while p-38 activation was curtailed after follow-up. In our previous study in pulmonary TB patients, we have shown that MTB antigens can modulate TCR and MAPK activation, which is crucial for protective immunity against TB. We also reported that MAPK activation is a crucial step in the TCR-mediated signaling cascade, leading to T-cell activation and, ultimately, a coordinated immune response against MTB in the context of pleurisy (28).

In the present study, we observed differential modulations in the activation of signaling pathway molecules in response to Ag85A and ESAT-6. They are the critical antigens to study TB immune responses due to their immunogenicity, specificity, and relevance to MTB pathogenesis. Their use in research and diagnostics provides insights into T-cell responses, cytokine production, and disease progression, making them indispensable tools to advance TB immunology and therapeutic development (38, 39).

In TB infection, IFN-γ production from T cells increases in response to increased TB antigenic burden, so a decline in IFN-γ concentrations might be assumed as a signal of a successful treatment response. A general decline in IFN-γ levels in response to treatment but with considerable variability in individual responses has been observed and with most patients still testing positive at the end of treatment (40). The frequency of IFN-γ-secreting cells declines during TB treatment, while the frequency of IL-2-secreting cells increases (41). In this study, we evaluated the frequency of IFN-γ- and IL-2-producing CD3+CD4+ T cells in TBP patients at baseline and after 6 months of follow-up. Our results show an increased percentage of IFN-γ- and IL-2-producing CD3+CD4+ T cells after treatment compared to baseline. The observed increased TCR-mediated ERK1/2 and p38 phosphorylation post-treatment in our findings might be the reflection of increased antigenic stimulation. Increased IFN-γ and IL-2 production post-treatment supports partial Th1 recovery, consistent with studies showing time-dependent Th1 cytokine restoration in treated TB patients (41, 42). However, the interplay between MAPK signaling, PKC-θ activation, and cytokine production requires further exploration, particularly in the context of immune exhaustion or regulatory T-cell activity post-treatment.

Cytokines are messengers that coordinate the development and function of T cells; specific plasma cytokines exhibit remarkable specificity in distinguishing various stages of TB, from latency to drug sensitivity and drug resistance (43, 44). However, the exact role of these cytokines in treatment outcomes remains inconclusive.

Recognition of MTB by phagocytic cells leads to cell activation and production of cytokines, which in itself induces further activation and cytokine production in the complex process of regulation and cross-regulation. The pro-inflammatory cytokine network plays a crucial role in inflammatory responses and the outcome of MTB infection. A study done in India showed significantly higher levels of IFN γ, TNF α, IL-17A, and IL-1β at baseline, and the plasma levels of all the cytokines examined were significantly reduced after successful chemotherapy (45).

During post-treatment in TB, many other factors such as T-cell exhaustion, altered memory T-cell responses, and regulatory T-cell (Treg) activity play significant roles, often contributing to the difficulty in completely eliminating the infection or leading to reinfection. T-cell exhaustion occurs when persistent antigen presentation leads to chronic T-cell stimulation. Chronically activated T cells undergo transcriptional changes, including increased co-inhibitory receptor (i.e., PD-1, CTLA-4, TOX, TIM-3, LAG-3, TIGIT) expression and decreased effector function (46). This impairs the ability of the immune system to clear the infection completely, contributing to treatment failure or reactivation. Even after treatment, some level of T-cell exhaustion may persist, limiting the sustained immune control necessary to prevent TB recurrence or reinfection (47). Almeida et al. found a correlation between increased immune activation and an increased frequency of Treg cells in patients who had completed treatment. They suggested that the presence of persisting immune activation and correspondingly high frequencies of regulatory T lymphocytes may reflect immune dysregulation that predisposes individuals to clinical tuberculosis, specifically to extrapulmonary TB (48). Effective TB control relies on a balance between effector and memory T cells. After acute infection, a small fraction of effector T cells matures into memory T cells, which provide long-term protection and rapid reactivation upon re-exposure. However, insufficient or dysfunctional memory T-cell populations could fail to provide sustained immunity, leading to the potential for reinfection (49).

Our findings of modulations in the activation of TCR-regulated T-cell signaling molecules in TBP patients after treatment offer a potential advancement in the field of TB management, enabling the early identification of individuals. Further research and validation studies are needed to explore their role in designing better and more efficient TB treatment strategies. Lastly, these findings would be useful in improving our ability to predict treatment outcomes to reduce the burden of TB and enhance the effectiveness of TB control programs worldwide.

There are some noteworthy limitations of this study; these limitations include the small cohort size and lack of longitudinal pleural fluid samples to compare local and systemic responses. Future studies should investigate TCR signaling in tissue-resident T cells and explore MAPK inhibitors as adjunctive therapies to modulate immune responses.

## Conclusion

This study proves that 6 months of anti-TB treatment modulates the activation of TCR-mediated signaling molecules in the blood of patients, reflecting changes in antigenic stimulation but suggesting persistent immune dysregulation. These findings highlight the need for targeted studies on the mechanism of T-cell signaling to inform host-directed therapies and prevent TB relapse. ATT enhances T-cell signaling and Th1 responses in TBP patients by upregulating TCR signaling. These immunological changes correlate with clinical resolution and highlight the therapeutic potential of targeting T-cell suppression for improved outcomes. Further research should focus on larger cohorts to validate these findings and explore their inferences for effective TB vaccine development and to explore strategies for better immunotherapeutic approaches.

## Data Availability

The raw data supporting the conclusions of this article will be made available by the authors, without undue reservation.
